# Enhanced rapid commercial DNA extraction kit for the molecular detection of severe acute respiratory syndrome coronavirus 2: Easy adaptation to current protocols

**DOI:** 10.1590/0037-8682-0270-2021

**Published:** 2021-11-12

**Authors:** Rômulo Pessoa-e-Silva, Priscilla Stela Santana de Oliveira, Sayonara Maria Calado Gonçalves, Klarissa Miranda Guarines, Lidiane Vasconcelos do Nascimento Carvalho, Maria Andreza Bezerra Correia, Michelle Melgarejo da Rosa, Moacyr Jesus Barreto de Melo Rêgo, Maira Galdino da Rocha Pitta, Michelly Cristiny Pereira

**Affiliations:** 1 Universidade Federal de Pernambuco, Núcleo de Pesquisa em Inovação Terapêutica - Suely Galdino, Recife, PE, Brasil.; 2 Universidade Federal de Pernambuco, Departamento de Fisiologia e Farmacologia, Recife, PE, Brasil.; 3 Universidade Federal de Pernambuco, Departamento de Bioquímica, Recife, PE, Brasil.

**Keywords:** COVID-19, SARS-CoV-2, Rapid extraction protocol, RT-qPCR

## Abstract

**INTRODUCTION:**

Herein, the authors describe a simple enhancement to a commercial rapid DNA extraction kit based on simple viral lysis for detecting COVID-19 via RT-qPCR.

**METHODS:**

After testing several different modifications, the adapted protocol with the best results in preliminary experiments was statistically evaluated in comparison with an automated robotic protocol.

**RESULTS:**

Processing and testing of 119 nasopharyngeal samples ultimately yielded near-perfect agreement with the automated protocol (κ = 0.981 [95% confidence interval 0.943-1.000]).

**CONCLUSIONS:**

The low cost and rapidity of the enhanced protocol makes it suitable for adoption in laboratories diagnosing COVID-19, especially those with high demand for examinations.

In late 2019, the emergence of a novel virus precipitated a global public health crisis. Severe acute respiratory syndrome coronavirus 2 (SARS-CoV-2), whose transmission began in Wuhan, China, was identified as the causative agent of coronavirus disease 2019 (COVID-19). By June 12, 2021, the virus had been registered in 222 countries and territories, and caused 3.14 million deaths worldwide^1^ ^,^ ^2^. 

Robust testing using reverse transcription followed by real-time quantitative polymerase chain reaction (RT-qPCR) ranks among the most used strategies adopted by health care entities and governments to monitor cases of COVID-19 and prevent the spread of the virus, primarily via the identification of asymptomatic individuals^3^ ^-^ ^6^. Extensive testing of the population must be performed using a reliable and accurate diagnostic protocol. Rapid DNA and RNA extraction protocols based on simple viral lysis are highly useful for increasing the testing capacity of clinical laboratories. However, these extraction procedures generally do not guarantee purity or sufficient removal of PCR inhibitors, which decreases their sensitivity and can lead to false-negative results^7^ ^-^ ^9^. In this article, we describe an adaptation to a commercial rapid extraction kit for COVID-19 detection via RT-qPCR, ensuring a sensitivity comparable to that of an automated commercial protocol. 

In total, five assays were performed sequentially (Figure 1). First, a preliminary test was performed with 20 nasopharyngeal swab samples collected for COVID-19 investigation and chosen at random. The aim of this preliminary test was to determine whether the rapid extraction protocol would be able to extract viral RNA of sufficient quality and quantity to detect SARS-CoV-2 in nasopharyngeal samples. Swabs were placed in phosphate-buffered saline (PBS, 4 mL). For processing, the tubes were vortexed, and RNA extraction was performed using two methods simultaneously (different aliquots): an automated procedure; and a rapid manual extraction procedure. The kits used were the Maxwell® RSC Viral Total Nucleic Acid kit (AS1330), for use in robotic extraction with the Maxwell RSC 48 Instrument (Promega, Madison, WI, USA) using magnetic beads (sample volume, 200 µL; elution volume, 50 µL of RNase-free water), and the QuickExtract^TM^ kit (Lucigen®, Middleton, WI, USA), using 20 µL of the DNA extraction solution 1.0 (Cat. No. QE09050) (sample volume, 20 µL; elution volume, 20 µL [1:1]). All procedures were performed according to manufacturer’s instructions. RT-qPCR was performed using the GoTaq® Probe 1-Step RT-qPCR System (Promega) and primers/probes 2019-nCoV_N1/N2/RP CDC (Centers for Disease Control and Prevention [CDC], Atlanta, GA, USA). All qPCR assays were run on a QuantStudio™ 5 Real-Time PCR System (Thermo Fisher Scientific, Carlsbad, CA, USA). For assay analysis, QuantStudio™ 3 and 5 systems version 1.5.1 were used.

**FIGURE 1:**
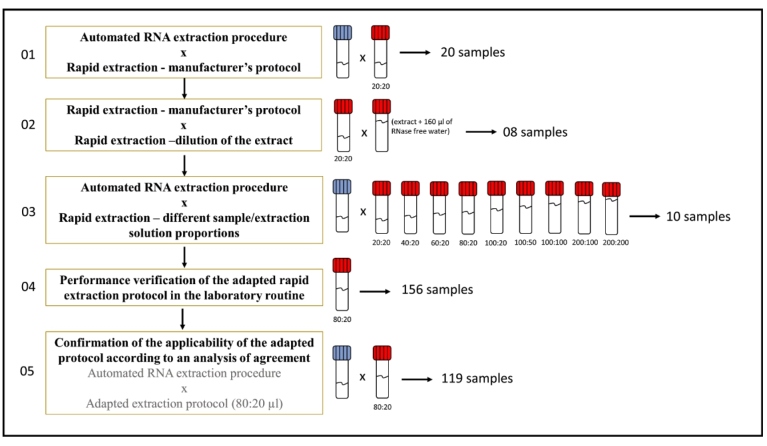
Schematic flowchart of the assays carried out to optimize the rapid extraction protocol and to verify its applicability in the routine to detect SARS-CoV-2 in nasopharyngeal samples by RT-qPCR. **Legend:** Tubes with blue cap represent samples extracted by the automated procedure, and tubes with red cap represent samples extracted by the rapid protocol.

For all experiments, samples were considered to be positive, negative, or inconclusive according to criteria established by the CDC^10^, as follows: positive, N1 and N2, cycle threshold (Ct) < 40.0; negative, N1 and N2, Ct ≥ 40.0, and endogenous control (RNase P [RP]) < 40.0; and inconclusive, amplification of only one target sequence (i.e., N1 or N2), Ct value < 40.0. 

 The results yielded 18 positive samples (90%) in the automated procedure, seven (38.89%) of which were positive using the rapid protocol. All samples extracted using both protocols yielded amplification of RP, with Ct values < 36.0. The rapid extraction protocol yielded viral RNA of sufficient quality and quantity to be detected in some samples. However, given the loss in sensitivity compared to the automated procedure, and the need for a quick and reliable extraction method in routine laboratory protocols, further tests were performed. 

In the next test, eight positive samples not included in the first test and with different Ct values in the automated procedure were extracted from a new aliquot (stored at -80°C) in duplicate using the rapid procedure. In one of the duplicates, 160 µL of RNase-free water was added to 40 µL of extracted sample (20 µL of sample + 20 µL of extraction solution) to evaluate possible interference from PCR inhibitors. Three samples with late Ct values (N1 and N2 > 32.0) were negative in both duplicates (with and without dilution). The remaining five samples were positive (Table 1). Based on these results, different proportions of sample and extraction solution volumes were tested in an attempt to improve the detection capability of the rapid protocol. Ten positive samples, all with late Ct values in the automated procedure (Ct > 32.0), were chosen. The samples were extracted from new aliquots stored at -80°C. The proportions of sample and extraction solution (all in µL) were evaluated, in addition to the 20:20 proportion recommended by the manufacturer: 40:20, 60:20, 80:20, 100:20, 100:50, 100:100, 200:100, and 200:200. In the 80:20 µL proportion, six samples (60%) remained positive, while in the 20:20 µL proportion recommended by the manufacturer, only four (40%) were positive. The results indicated that the 80:20 µL proportion demonstrated the best performance in RT-qPCR. All other conditions yielded ≤ 5 positive samples. 

**TABLE 1:** Results of RT-qPCR for SARS-CoV-2 detection in nasopharyngeal swab samples extracted with a rapid DNA extraction protocol with and without dilution of the extracted sample and with an automated RNA extraction protocol.

Sample	Automated RNA			Rapid extraction -			Rapid extraction (dilution of the		
(nº)	extraction			manufacturer’s			extracted sample: 160 µL of		
	procedure			protocol			RNase free water)		
	N1	N2	RP	N1	N2	RP	N1	N2	RP
1	17.91	18.22	25.11	21.95	25.77	31.59	22.02	25.65	29.25
2	16.67	16.46	24.52	20.26	23.40	30.46	22.50	25.38	32.94
3	25.53	27.26	24.04	30.93	35.36	28.36	32.42	36.27	31.26
4 (late Ct)	32.37	34.36	25.58	(-)	(-)	31.82	(-)	(-)	32.95
5	19.68	19.95	25.11	24.32	28.04	30.18	26.47	29.83	32.75
6	20.22	20.76	27.28	24.22	23.67	31.14	26.91	26.50	34.2
7 (late Ct)	36.77	34.42	26.85	(-)	(-)	32.00	(-)	(-)	35.95
8 (late Ct)	35.63	39.82	25.33	(-)	(-)	28.57	(-)	(-)	32.01
**Mean Ct value**	**25.59**	**26.40**	**25.47**	**24.33**	**27.24**	**30.51**	**26.06**	**28.72**	**32.66**

**Legend: Ct:** Cycle threshold. **(-):** no amplification of the target. A late Ct was considered when Ct value > 32.0.

Thereafter, to verify the performance of the adapted rapid extraction protocol in the routine laboratory protocol, 156 samples were randomly selected, with all swabs placed in PBS, and extracted using the optimal proportion (i.e., 80:20 µL). As a result, 100 samples were negative and, of the remaining 56, five were inconclusive. Samples that were negative or inconclusive were re-extracted from a new aliquot (stored at -80°C) using an automated procedure. After re-extraction, the five inconclusive samples tested positive, whereas the other 100 samples remained negative. These results indicated that no false-negative results were obtained after using the modified rapid extraction protocol.

Finally, to confirm the applicability of the adapted protocol according to analysis of agreement, 119 nasopharyngeal swab samples, all stored in PBS, were randomly selected and extracted using the automated procedure and the rapid protocol. After RT-qPCR and data analysis, the automated extraction protocol identified 52 positive and 67 negative samples, as with the adapted rapid protocol, 41 positive, 68 negative, and 10 inconclusive samples were obtained. Considering only positive and negative samples, 99.08% agreement between the extraction protocols was observed. Calculation of the kappa (κ) coefficient revealed very good agreement (κ = 0.981 [95% confidence interval 0.943-1.000]), as shown in Table 2 ^11^. The mean Ct values obtained for the 52 positive samples were similar between the extraction protocols for both targets: automated procedure, N1 = 26.17, N2 = 27.45; adapted rapid protocol, N1 = 26.96, N2 = 31.54. It is important to highlight that the RP of all 119 samples extracted using both extraction protocols amplified with Ct values not exceeding 36.0. Mean Ct values for RP: automated procedure, 27.36; adapted rapid protocol, - 28.84 ( Table 3, Supplementary Material). 

**TABLE 2:** Comparison of the automated RNA extraction protocol and the adapted rapid DNA extraction protocol.

		Automated extraction procedure (Robotic extraction)		
		Positive	Negative	Total
Adapted rapid DNA extraction protocol (20/80)	Positive	41	0	**41**
	Negative	1	67	**68**
**Total**		**42**	**67**	**109**
Kappa		0.981 (very good)		
SE		0.019		
CI (95%)		0.943 - 1.000		

Legend: **SE:** Standard error; **CI:** confidence interval.

The results highlight that the adapted rapid extraction protocol was as sensitive as the automated protocol, ultimately reflected by near-perfect agreement. This finding reinforces the applicability of the rapid protocol in routine laboratory procedures for detecting COVID-19 using RT-qPCR. The rapid protocol requires only heat treatment of the sample mixed with an extraction solution to lyse the virus and release the genomic content. Previous studies have demonstrated the possibility of directly detecting SARS-CoV-2 via RT-qPCR by using only heated and/or lysed samples and without any significant loss in detection capability^12^ ^-^ ^14^. Furthermore, in addition to its speed and practicality, the cost per patient of the rapid extraction kit can be 20 times less than that of the automated extraction kit (prices based on quotes from December 2020). 

These characteristics make the quick protocol suitable for adoption in laboratories that diagnose COVID-19, especially those with a high demand for examinations such as central reference and public health laboratories. Robotic extraction can be used specifically to retest samples with inconclusive results. 

In this study, all tests were performed using nasopharyngeal swabs soaked in PBS. For samples in viral transport medium or other types of media, however, it is critical to execute an appropriate test before using the adapted rapid protocol due to the possible presence of PCR inhibitors. 

The adaptation proposed in the tested protocol-given its simplicity and efficiency-can be used to improve the sensitivity of rapid extraction kits from different manufacturers. If an automated/robotic extraction procedure is not accessible, the rapid protocol can be compared using column-based RNA extraction kits available in the laboratory. Moving forward, it is mandatory to critically review all stages of any extraction protocol in use and to perform statistical comparisons with different extraction kits to confirm its applicability before use. 
