# Supplementary material for: Enhanced rapid commercial DNA extraction kit for the molecular detection of severe acute respiratory syndrome coronavirus 2: Easy adaptation to current protocols
Source: Rev Soc Bras Med Trop. 2021 Nov 12;54:e0270-2021. doi: 10.1590/0037-8682-0270-2021 (PMC8582969; doi:10.1590/0037-8682-0270-2021)
Supplement: Supplementary file 1 [file 1678-9849-rsbmt-54-e0270-2021-supp1.pdf]

TABLE 3: COVID-19 RT-qPCR results obtained from nasopharyngeal samples after RNA extraction with the automated and the adapted rapid extraction protocols.

	Rapid extraction - adapted protocol - 80:20 µl			Automated RNA extraction procedure		
**Sample code**	**N1**	**N2**	**RP**	**N1**	**N2**	**RP**
**AMU19242**	(-)	(-)	25,668	(-)	(-)	24,475
**AMU19243**	(-)	(-)	27,326	(-)	(-)	26,075
**AMU19244**	(-)	(-)	28,305	(-)	(-)	25,655
**AMU19245**	(-)	(-)	29,851	(-)	(-)	26,764
**AMU19246**	24,811	30,071	25,847	21,348	23,461	24,173
**AMU19247**	32,288	39,259	24,29	27,284	29,62	24,161
**AMU19248**	30,219	36,045	27,038	21,222	23,718	24,595
**AMU19249**	27,606	41,014	28,671	25,926	28,247	27,226
**AMU19250**	(-)	(-)	31,688	(-)	(-)	29,81
**AMU19251**	(-)	(-)	32,587	(-)	(-)	30,431
**AMU19252**	33,656	Und	27,963	30,199	32,915	26,737
**AMU19253**	(-)	(-)	26,256	(-)	(-)	25,428
**AMU19254**	19,676	27,584	30,049	19,567	21,614	28,445
**AMU19255**	(-)	(-)	28,701	(-)	(-)	27,572
**AMU19256**	(-)	(-)	27,61	(-)	(-)	24,876
**AMU19257**	31,665	36,967	30,588	30,316	32,698	27,428
**AMU19258**	19,313	25,776	27,899	17,9	19,327	26,765
**AMU19259**	29,146	34,43	27,869	26,721	28,96	27,243
**AMU19260**	(-)	(-)	27,073	(-)	(-)	27,908
**AMU19261**	(-)	(-)	29,285	(-)	(-)	27,45
**AMU19262**	29,674	34,442	28,33	27,557	28,98	26,869
**AMU19263**	25,611	30,887	28,074	23,976	24,653	26,944
**AMU19264**	22,466	27,605	28,235	20,678	21,667	26,846
**AMU19265**	19,046	23,467	28,019	18,011	18,571	27,99
**AMU19266**	(-)	(-)	29,741	(-)	(-)	28,559
**AMU19267**	(-)	(-)	26,647	(-)	(-)	26,8709
**AMU19268**	(-)	(-)	26,973	(-)	(-)	28,004
**AMU19269**	(-)	(-)	25,983	(-)	(-)	26,54
**AMU19270**	(-)	(-)	30,015	(-)	(-)	29,312
**AMU19271**	19,444	26,013	31,444	17,827	19,049	27,752
**AMU19272**	(-)	(-)	33,004	(-)	(-)	34,019
**AMU19273**	(-)	(-)	30,551	(-)	(-)	30,901
**AMU19274**	19,143	25,001	27,41	19,261	20,559	26,502
**AMU19275**	31,069	38,849	29,692	35,289	36,977	33,828
**AMU19276**	30,021	36,813	32,483	30,383	32,374	32,185
**AMU19277**	19,66	24,596	28,159	20,305	19,812	28,732

Continue...

**AMU19278**	(-)	(-)	30,387	(-)	(-)	29,44323
**AMU19279**	18,269	23,804	30,467	17,909	18,156	29,393
**AMU19280**	32,766	43,175	32,67	32,639	33,075	31,018
**AMU20084**	19,346	22,899	29,060	18,828	19,154	28,176
**AMU20085**	28,390	33,166	28,274	26,949	27,829	28,151
**AMU20086**	23,483	27,099	29,034	22,084	23,262	27,957
**AMU20087**	28,993	33,354	28,108	28,115	31,244	25,868
**AMU20088**	(-)	(-)	30,559	(-)	(-)	28,531
**AMU20089**	34,395	40,285	29,675	34,433	34,496	27,308
**AMU20090**	31,576	37,897	26,053	30,083	30,763	24,561
**AMU20091**	(-)	(-)	30,976	(-)	(-)	29,071
**AMU20092**	(-)	(-)	33,543	(-)	(-)	30,969
**AMU20093**	(-)	(-)	33,292	(-)	(-)	30,743
**AMU20094**	(-)	(-)	29,762	42,791	(-)	27,241
**AMU20095**	22,169	25,854	28,832	(-)	(-)	14,163
**AMU20096**	22,107	25,748	26,086	(-)	(-)	13,451
**AMU20597**	(-)	(-)	23,743	(-)	(-)	23,128
**AMU20598**	(-)	(-)	35,583	(-)	(-)	29,47
**AMU20599**	36,216	(-)	34,581	26,32	28,847	28,775
**AMU20600**	36,272	40,57	33,185	27,364	29,897	30,365
**AMU20601**	(-)	(-)	32,979	(-)	(-)	28,697
**AMU20602**	(-)	(-)	32,411	(-)	(-)	29,303
**AMU20603**	19,223	21,971	24,405	16,866	18,838	26,478
**AMU20604**	(-)	(-)	31,972	(-)	(-)	28,822
**AMU20605**	(-)	(-)	27,93	(-)	(-)	28,952
**AMU20606**	23,813	33,063	34,116	20,723	22,539	31,435
**AMU20607**	27,689	32,358	30,656	25,843	28,931	28,34
**AMU20608**	(-)	(-)	31	(-)	(-)	31,771
**AMU20609**	(-)	(-)	29,76	(-)	(-)	29,736
**AMU20610**	(-)	(-)	27,792	(-)	(-)	28,722
**AMU20611**	(-)	(-)	27,452	(-)	(-)	28,115
**AMU20612**	(-)	(-)	23,056	(-)	(-)	24,677
**AMU20613**	(-)	(-)	28,416	36,84	38,967	29,417
**AMU20614**	(-)	(-)	19,586	(-)	(-)	23,283
**AMU20615**	29,766	33,574	25,016	30,204	33,525	26,396
**AMU20616**	12,804	14,09	24,382	14,425	15,659	25,714
**AMU20617**	33,088	37,235	29,397	28,126	29,743	28,159
**AMU20618**	36,876	40,635	30,747	32,454	34,795	30,103
**AMU20619**	20,48	24,487	28,427	24,675	26,286	31,458
**AMU20620**	(-)	(-)	29,301	(-)	(-)	28,64
**AMU20621**	38,194	(-)	30,966	36,448	39,965	30,004
**AMU20622**	(-)	(-)	29,241	(-)	(-)	29,229
**AMU20623**	(-)	(-)	30,558	(-)	(-)	31,566

Continue...

**AMU20624**	(-)	(-)	30,766	(-)	(-)	30,973
**AMU20625**	27,499	33,609	29,719	26,865	28,303	28,489
**AMU20626**	(-)	(-)	30,477	(-)	(-)	31,171
**AMU20627**	(-)	(-)	24,566	(-)	(-)	24,759
**AMU20628**	(-)	(-)	25,758	(-)	(-)	25,993
**AMU20629**	(-)	(-)	27,543	(-)	(-)	29,158
**AMU20630**	(-)	(-)	29,53	(-)	(-)	29,925
**AMU20631**	(-)	(-)	24,523	(-)	(-)	27,486
**AMU20632**	(-)	(-)	29,197	(-)	(-)	29,112
**AMU20633**	(-)	(-)	30,436	(-)	(-)	29,128
**AMU20634**	(-)	(-)	30,034	(-)	(-)	30,566
**AMU20635**	(-)	(-)	25,472	(-)	(-)	26,278
**AMU20636**	(-)	(-)	29,311	(-)	(-)	28,015
**AMU20637**	(-)	(-)	29,306	42,474	(-)	25,113
**AMU20638**	(-)	(-)	33,328	(-)	(-)	29,453
**AMU20639**	(-)	(-)	29,477	(-)	(-)	25,885
**AMU20640**	29,751	34,025	29,728	28,129	29,838	26,894
**AMU20641**	(-)	(-)	29,674	(-)	(-)	26,547
**AMU20642**	29,681	41,994	29,834	28,057	28,611	26,563
**AMU20643**	(-)	(-)	26,423	(-)	(-)	23,053
**AMU20644**	32,959	40,204	29,258	30,826	31,521	25,034
**AMU20645**	24,124	27,081	32,254	21,397	23,63	28,805
**AMU20646**	22,744	25,867	28,975	19,557	25,848	25,659
**AMU20896**	(-)	(-)	29,846	(-)	(-)	26,834
**AMU20897**	(-)	(-)	27,957	(-)	(-)	25,027
**AMU20898**	(-)	(-)	31,478	(-)	(-)	28,976
**AMU20899**	(-)	(-)	28,642	(-)	(-)	25,542
**AMU20900**	(-)	(-)	27,492	(-)	(-)	25,123
**AMU20901**	(-)	(-)	28,966	(-)	(-)	25,527
**AMU20902**	(-)	(-)	29,123	(-)	(-)	24,498
**AMU20903**	(-)	(-)	28,063	(-)	(-)	26,848
**AMU20904**	(-)	(-)	29,09	(-)	(-)	25,36
**AMU20905**	(-)	(-)	28,525	(-)	(-)	26,109
**AMU20906**	(-)	(-)	28,742	(-)	(-)	25,96
**AMU20907**	35,095	38,478	24,759	34,251	36,221	23,105
**AMU20908**	29,604	32,307	26,84	27,846	29,048	25,171
**AMU20909**	(-)	(-)	25,57	(-)	(-)	24,317
**AMU20910**	27,146	29,869	26,385	25,361	26,528	24,472
**AMU20911**	29,18	32,654	29,125	28,063	30,08	26,986
**AMU20912**	23,44	26,449	28,25	21,394	22,494	26,483
**AMU20913**	27,437	27,437	29,576	24,43	25,748	25,369
**AMU20914**	22,675	25,213	26,603	20,619	22,135	25,372
**AMU20915**	(-)	(-)	26,028	(-)	(-)	25,63

Legend: (-): no amplification of the target. AMU and COV are the laboratory codes to distinguish samples according to their origin: Metropolitan Region of Recife (COV) or the Interior of Pernambuco state (AMU), Brazil.
